# Freeze Granulated Zeolites X and A for Biogas Upgrading

**DOI:** 10.3390/molecules25061378

**Published:** 2020-03-18

**Authors:** Kritika Narang, Farid Akhtar

**Affiliations:** Division of Materials Science, Luleå University of Technology, 97187 Luleå, Sweden; kritika.narang@ltu.se

**Keywords:** freeze granulation, zeolite NaX, zeolite CaA, gas separation, carbon dioxide capture

## Abstract

Biogas is a potential renewable energy resource that can reduce the current energy dependency on fossil fuels. The major limitation of utilizing biogas fully in the various applications is the presence of a significant volume fraction of carbon dioxide in biogas. Here, we used adsorption-driven CO_2_ separation using the most prominent adsorbents, NaX (faujasite) and CaA (Linde Type A) zeolites. The NaX and CaA zeolites were structured into hierarchically porous granules using a low-cost freeze granulation technique to achieve better mass transfer kinetics. The freeze granulation processing parameters and the rheological properties of suspensions were optimized to obtain homogenous granules of NaX and CaA zeolites 2–3 mm in diameter with macroporosity of 77.9% and 68.6%, respectively. The NaX and CaA granules kept their individual morphologies, crystallinities with a CO_2_ uptake of 5.8 mmol/g and 4 mmol/g, respectively. The CO_2_ separation performance and the kinetic behavior were estimated by breakthrough experiments, where the NaX zeolite showed a 16% higher CO_2_ uptake rate than CaA granules with a high mass transfer coefficient, 1.3 m/s, compared to commercial granules, suggesting that freeze-granulated zeolites could be used to improve adsorption kinetics and reduce cycle time for biogas upgrading in the adsorption swing technology.

## 1. Introduction

Biogas offers a sustainable fuel to the environment as it contributes to low carbon emissions during its combustion. The raw biogas obtained from an anaerobic digester mainly consist of CH_4_ with 40–45% CO_2_ including a minor content of impurities, such as water, siloxanes, ammonia and hydrogen sulfide [1,2]. Great efforts have been made in the biogas separation and purification technologies to procure biomethane with a low energy footprint [3,4]. The removal of CO_2_ from biogas increases the methane content to above 98 vol%, which offers the use of upgraded biogas in natural gas grids and as a fuel for automotive industry [5]. The pressure swing adsorption technology (PSA) is one of the promising technologies for gas separation and production of gases used by industries [6,7,8]. In the context of biogas upgrading using the PSA technology, the structured adsorbent in the form of granules, pellets, laminates, and extrudates of zeolites, microporous silicates and activated carbon are utilized to separate CO_2_ from the biogas stream by exploiting equilibrium, kinetic, as well as size-selective adsorption strategies [9,10,11]. Zeolites are well-studied kinetically and thermodynamically for their CO_2_ separation from CH_4_ and N_2_, as they provide high volumetric CO_2_ adsorption capacity even at low CO_2_ partial pressure [12,13]. Zeolites X, A and chabazite offer promising properties, such as remarkable selectivity of CO_2_ over CH_4_, high thermal stability, fast adsorption kinetics, and cyclic performance, which together reduce the energy penalty in the separation and purification process [14,15,16].

The NaX zeolite has a faujasite (FAU) type framework with sodalite cages comprised of SiO_4_ and AlO_4_ tetrahedra and connected through double six-rings to create a large super-cage cavity (aperture 0.74 nm) [17]. The CaA zeolite has a Linde type A (LTA) structure where sodalite cages are connected through double four-rings and give rise to an alpha cavity (aperture 0.43 nm) [17].

Zeolites are most often produced in the powder form; however, powder offers challenges, mainly in the pressure swing adsorption technology during the biogas cleaning-up process. Using porous materials in the powder form offers significant drawbacks, such as fluidization of the PSA column and high particle attrition rate, which further give rise to a high pressure drop within the column and thereby an increase in the maintenance cost and energy consumption of the PSA systems in addition to poor adsorption and regeneration of the adsorbent material [18,19]. Hence, there remains a need to structure the zeolite powders into mechanically stable hierarchically porous structures, such as granules, monoliths, honeycombs, laminates, etc., with favorable carbon capture kinetics to permit efficient CO_2_ separation from biogas. The structured zeolites provide high mass transfer, low pressure drop, better heat management in the adsorption columns in the PSA [18,20]. For instance, Wenjing Zhang et al. have designed hierarchical composite ZSM-5 pellets for biogas upgrading and achieved a 30% larger BET surface area with a 34% increase in CO_2_ uptake capacity for the hierarchically structured porous zeolite [21].

Structuring of the zeolites into granules and beads is a simple and facile approach for improving flowability, mechanical strength, CO_2_ separation performance, rapid mass transfer kinetics, and to facilitate high CO_2_ uptake. The small granule size is preferred to obtain high mass transfer kinetics with low void fraction to improve the carbon-capturing efficiency; however, small size granules also give rise to a high pressure drop in the PSA unit [18,22,23]. Therefore, granules’ characteristics must be optimized in a way to facilitate control over granular size, density, and macroporosity. In recent years, reverse wet granulation, foam granulation, thermal adhesion granulation, spray drying, freeze granulation, and sieve granulation have been used to granulate powders [18,24]. Freeze granulation is a recently developed method for granulation that has shown ideal characteristics of the granulation process by providing homogeneous spherical granules with a uniform size and improved flowability. Freeze granulation process provides a versatile approach in introducing hierarchical porous structures with the ability to control pore size, morphology, and orientation. Control over the freezing rate can offer modulation in ice-templating during the freeze granulation process to provide high open porosity in the structured adsorbents [25]. It is beneficial to use the freeze-drying process as the ice sublimes leaving structured material with continuous residual ice template pores and results in homogenous granules. Freeze granulation has previously been used to process metal, metal oxide, and ceramic powders industrially for applications as transparent ceramics and in the pharmaceutical sector [26]. Suitable inorganic and organic binders are used to increase the mechanical strength of the granules. Raksh V. Jasra et al. have shown the effect of binders on the sorption properties of Y and mordenite zeolites and observed a 34−78% increase in the heat of adsorption for N_2_ in the respective structured zeolites [27]. Similarly, Charkhi Amir et al. have studied the effects of bentonite on the adsorption properties of the granulated NaY nanozeolite and showed that the increase in the bentonite content in the zeolite led to the reduction in the BET surface area [28]. Therefore, the type and amount of the binder can crucially affect the rheological characteristics of the suspension and the adsorptive properties of the structured zeolite [29]. Recently, M. Ouadaker et al. have prepared the porous granules of the Pickering emulsions stabilized with the halloysite mineral. They investigated such characteristics of the porous granules as morphology, size stability, strength, and porosity by varying the pH and ionic strength of the suspensions, and also showed that addition of organic binders, such as polyvinyl alcohol and polyethylene glycol, increases cohesion of the emulsions and leads to highly structured materials [29].

In the freeze granulation process, granules are instantly frozen in liquid nitrogen (−196 °C) and subjected to the freeze-drying process. This fast freezing process prevents migration of the constituents due to the limitations of the diffusion dynamics, thereby permitting production of homogenously structured granules. Migration of the constituents in the granulation process has been observed in other granulation techniques, such as spray drying, and it leads to non-homogeneous granules [30]. Furthermore, water-templating during the freeze granulation process creates additional macroporosity to offer additional mass transfer pathways to achieve superior mass transfer kinetics in the adsorption/desorption process [25,31,32]. Lyckfeldt et al. have shown that freeze granulation is a suitable alternative to the conventional granulation for processing the Si_3_N_4_ materials into spherical and free-flowing granules [33]. S.J. Milne studied the influence of different drying conditions on the powder properties and processing characteristics and concluded that freeze-drying led to a lower degree of agglomeration [34]. The research on the granulation of zeolites to produce hierarchical zeolite granules with optimum properties is still sparse. However, in this study, we have freeze-granulated NaX and CaA zeolites to investigate their carbon-capturing properties as regards to the CO_2_ and CH_4_ gas mixture.

In this work, we have optimized the design of highly porous granules with a hierarchy of pores from zeolite NaX and CaA powders using a low-cost robust freeze granulation approach, and evaluated the granules in terms of CO_2_ separation from CH_4_. Prior to the freeze granulation process, the zeolite suspension characteristics were investigated. The stable colloidal zeolite suspensions consisting of zeolite powder, bentonite clay, polyethylene glycol, polyacrylic acid, and water were prepared, and the optimum stirring rate was set up following the rheology data to achieve feasible viscosity for freeze granulation of highly porous granules. The granules were investigated for their CO_2_ and CH_4_ adsorption characteristics with their mass transfer kinetics.

## 2. Results and Discussion

**Figure 1:**
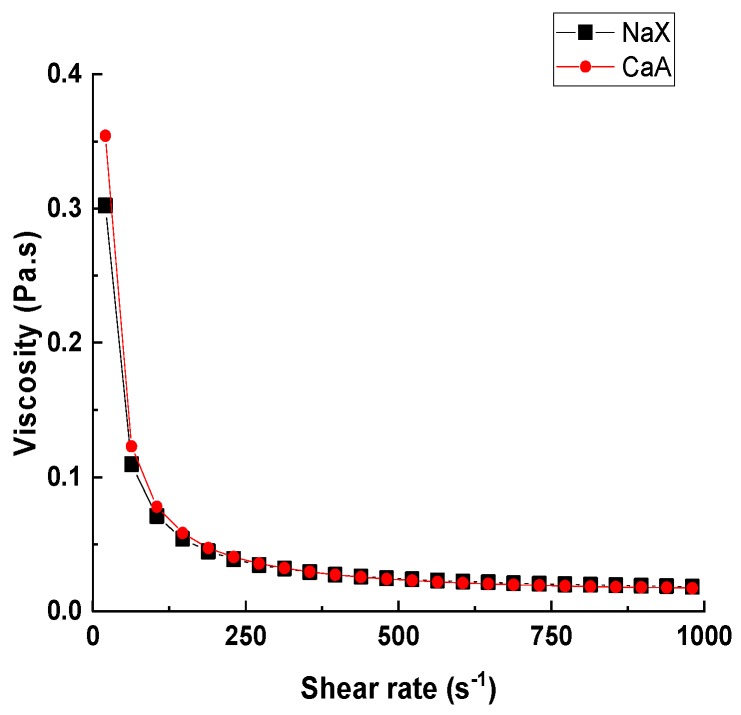
Viscosity as a function of the shear rate of the NaX suspension (black) and the CaA suspension (red) containing zeolite, bentonite, PEG (polyethylene glycol), PAA (polyacrylic acid), and water.

The granules of zeolites NaX and CaA, 2–3 mm in diameter were successfully produced using the freeze granulation process. The rheological behavior of the deagglomerated NaX and CaA suspensions prior to freeze granulation is shown in Figure 1. The solid content of the suspensions was calculated to obtain 70% macroporosity from water-templating in the granules. It can be seen in Figure 1 that zeolite suspensions exhibit the shear thinning behavior; the apparent viscosity decreases with the increase in the shear rate. It has been reported that zeolite-water suspensions show the Newtonian behavior, and it changes to the non-Newtonian one on changing either the pH of the suspension or with the addition of clay particles with the plate-like morphology [35,36,37]. In this work, the shear thinning behavior is due to the presence of the inorganic clay binder bentonite [35]. Bentonite is a widely available clay binder for the zeolites with wet binding characteristics to structure zeolites in hierarchical porous advanced materials. The suspension shows colloidal stability when the shear rate increases to and over 250 s^−1^. The colloidal stability of the zeolite-bentonite suspension was achieved due to the presence of the negative surface charge on the bentonite, as well as on zeolites NaX and CaA at the alkaline pH of 9.6. The pH of the formed suspensions was alkaline and gave rise to the negative surface charge, as the isoelectronic points of bentonite and zeolite are 8.0 and 4.7, respectively; this ensures electrostatically stabilized suspensions with good electrostatic dispersion of the particles [18,38,39].

To achieve a feasible flow for the granulation procedure, the shear rate of 355 s^−1^ was considered for the NaX suspension, as well as for the CaA suspension, and the stirring rate was calculated according to Equation (1) [40].

(1) $$\gamma =4*\pi *n/\left(1-k^{2}\right)$$

where the shear rate is related to the stirring frequency n and the geometric parameters of the stirring setup k, which is defined as the ratio between the magnetic stirrer length and the diameter of the beaker used. The magnetic stirrer length and cylindrical beaker diameter were 4.2 cm and 5 cm, respectively. The stirring frequency was calculated to be 500 rpm for both the suspensions to ensure a feasible flow and dispersibility of suspensions in the granulation process.

**Figure 2:**
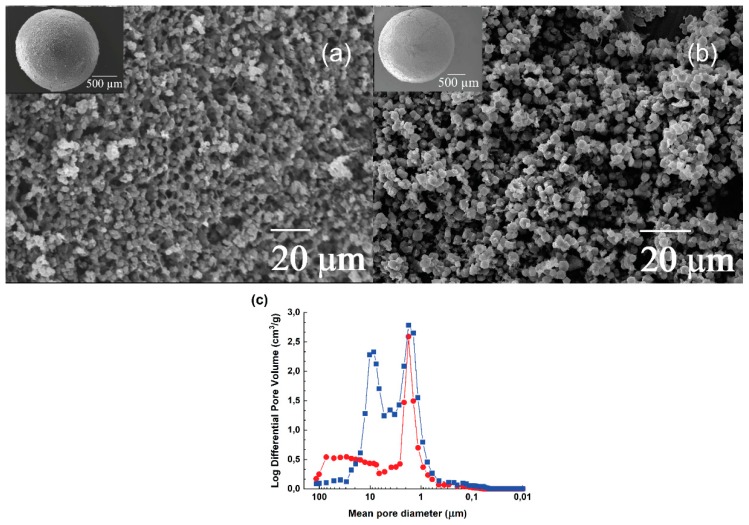
(**a**) Scanning electron micrograph of NaX granules with the inset of a single NaX granule; (**b**) Scanning electron micrograph of CaA granules with the inset of a single CaA granule; (**c**) Mercury intrusion porosimetry data for NaX granules (blue, square) and CaA granules (red, circle).

The morphology of the NaX and CaA zeolite granules with the inset of the whole granule is presented in Figure 2a,b. The granules exhibit spherical shape and possess a high degree of macroporosity (77.9% for NaX granules and 68.6% for CaA granules) determined using mercury intrusion porosimetry in Figure 2c, with a zeolite particle size from 2.5 to 4.5 microns for NaX granules and 2.5 to 4.0 microns for CaA granules. After the heat treatment, the original morphology of both the zeolite particles was intact, which implies that neither granulation nor freeze-drying or thermal treatment has any significant influence on the morphology of the starting zeolites.

The CaA granules show the pore size with the mean pore diameter of 1.7 µm, while NaX granules show bimodal pore size distribution with the mean pore diameter of 1.7 µm and 8.6 µm (Figure 2c). The mean pore diameter of 1.7 µm for both NaX and CaA granules describes the inter-pore distance between particles within the granules. In the case of NaX granules, the mean pore diameter of 8.6 µm obtained by ice-templating implies the existence of large inter-particle voids between the NaX particles within the granules formed during freeze-drying, which involves the sublimation of ice leaving behind large ice-template voids. Mercury intrusion porosimetry data show good agreement with the SEM micrographs presented in Figure 2a,b, which also display large inter-particle voids between the particles. The CaA granules showed ice-templating pores with a broad pore size distribution from ca. 6.5 to 102 µm. The reason for this broad distribution might be associated with the significant aeration in the CaA suspensions associated with a larger external surface area (Table 1), which ultimately leads to these large voids with trapped air in them.

**Figure 3:**
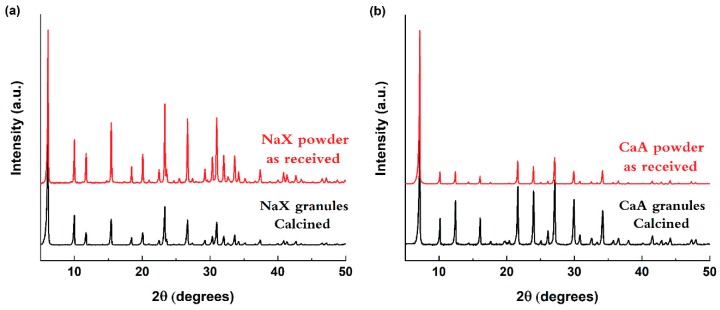
(**a**) XRD pattern of the NaX powder (above, red) and NaX granules (below, black); (**b**) XRD pattern of the CaA powder (above, red) and CaA granules (below, black). The NaX granules pattern matched the Faujasite (FAU) type framework, PDF card no. 00-0380237, and CaA granules pattern matched the Linde Type A (LTA), PDF card no. 01-073-9561.

Structuring the granules from the freeze granulation process followed by pressureless thermal treatment does not affect the crystal structure as verified by XRD patterns of calcined NaX and CaA granules in Figure 3a,b. The XRD patterns of granules correspond well with the XRD pattern of the starting zeolite powder, indicating that the freeze granulation process as well as the thermal treatment at 700 °C keep the crystallinity of the structured zeolites.

The textural properties of the zeolite NaX and CaA granules and powders are summarized in Table 1. The specific BET surface area has shown a decrease of 18.6% for the NaX granules and 22% for the CaA granules as compared to the starting zeolite powders. The active component in the fabricated granules is diluted due to the presence of an inactive bentonite binder, thereby resulting in a decrease in specific surface area. Another reason for this reduction is attributed to the blocking of micropores by the bentonite clay on binding zeolite crystals, which results in the lowering of adsorbed N_2_ per gram of granules. According to Amir Chakri et al., the increment of bentonite clay from 20 to 40 wt% in the granulated NaY nanozeolite can enhance the blocking of micropores and decrease the BET surface area by 66% [28]. The external surface area of the CaA powder is greater than of the NaX powder, which supports the hypothesis of a considerably higher aeration in the CaA suspension.

**Figure 4:**
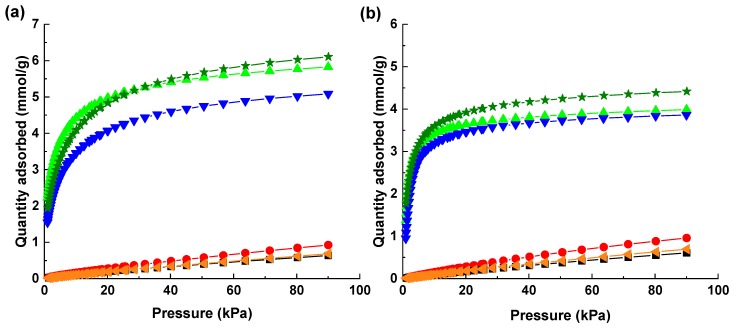
(**a**) CO_2_ adsorption isotherm (top) for NaX: for granules at 273 K (triangles) and at 293 K (inverted triangles), for the powder at 293 K (stars); CH_4_ adsorption isotherm (bottom) for NaX: for granules at 273 K (circles) and at 293 K (squares), for the powder at 293 K (rotated triangles); (**b**) CO_2_ adsorption isotherm (top) for CaA: for granules at 273 K (triangles) and at 293 K (inverted triangles), for the powder at 293 K (stars); CH_4_ adsorption isotherm (bottom) for CaA: for granules at 273 K (circles) and at 293 K (squares), and for the powder at 293 K (rotated triangles).

Figure 4 represents the adsorption isotherms of CO_2_ and CH_4_ for both NaX and CaA granules at 273 K and 293 K. The CO_2_ adsorption isotherm falls into the IUPAC type I isotherm [41] indicating that the material features microporosity. Zeolites NaX and CaA have high CO_2_ adsorption capacity due to the strong electrostatic interactions of the surface of the ionic aluminosilicate micropore cages and the quadrupole moment of CO_2;_ whereas at the same time, the CH_4_ adsorption isotherm is a linear isotherm showing a weak interaction between the zeolite and the CH_4_ due to the absence of the quadrupole moment resulting in low adsorption capacities [42]. The CO_2_ uptake capacity increases from 5.09 to 5.80 mmol/g from 293 K to 273 K, respectively, for the NaX granules. As the adsorption process is exothermic, a decrease in temperature will provide insufficient kinetic energy to gas molecules to desorb from the adsorbent, therefore, reduction in temperature enhances the uptake capacity. Furthermore, it was noticed that the difference in the CO_2_ uptake capacities for the CaA granules at 293 K and 273 K is not significant. The methane uptake was almost similar for both the granules. NaX granules have a higher CO_2_ uptake than CaA granules, and their corresponding powder zeolites show the same trend of adsorption as shown in Figure 4. Zeolite NaX powders have a higher CO_2_ uptake capacity than the zeolite CaA powder due to the higher specific BET surface area and pore volume of zeolite NaX [43]. The CO_2_ uptake capacity at 293 K of NaX and CaA granules is lower than their respective powders as shown in Figure 4. The zeolite particles in the granules contain an inorganic binder to impart additional strength to the granules. However, the binder dilutes the active component (zeolite) in granules per unit mass as compared to the pure NaX and CaA powders; this corresponds to the reduction in the CO_2_ adsorption capacity. Similar results were shown by Ojuva A. et al., where zeolite 13X powders were structured into monoliths using freeze casting to provide hierarchical porosity and observed a similar reduction in the CO_2_ adsorption capacity due to the presence of an inorganic binder [35].

The CO_2_ uptake capacity for the NaX and CaA granules is higher than the previously reported 3D-printed NaX and CaA zeolite monoliths [44]. The CO_2_ uptake of freeze-granulated NaX was 14% higher than of the structured NaX beads as reported by Fateme Rezaei et al. [45]. Similarly, the CO_2_ uptake of freeze-granulated CaA was 100% higher than of the granulated CaA zeolite reported by Leonel G. et al., where the granulation approach used was nucleation and further consolidation [46]. The CO_2_ adsorption capacity of the freeze-granulated NaX zeolite is comparable to the NaX monolith prepared by freeze casting [35]. Furthermore, the CO_2_ separation performance was investigated by determining the CO_2_-over-CH_4_ selectivity using the Henry’s law. NaX granules have a higher CO_2_-over-CH_4_ selectivity of 214, whereas CaA granules showed the CO_2_-over-CH_4_ selectivity of 172 at 273 K. It is established that selectivity and gas adsorption characteristics of zeolites can be tailored using ion exchange in zeolites [43]. Studies on the cation exchange from Na^+^ to Ca^2+^ in the X zeolite, or from Ca^2+^ to Na^+^ in the A zeolite have been conducted to tune the gas adsorption capacity and selectivity [47,48,49]. S. Chen et al. have recently shown the increase in the CO_2_ uptake after the ion exchange of Na^+^ cations with Ca^2+^ cations at different concentrations [50].

**Figure 5:**
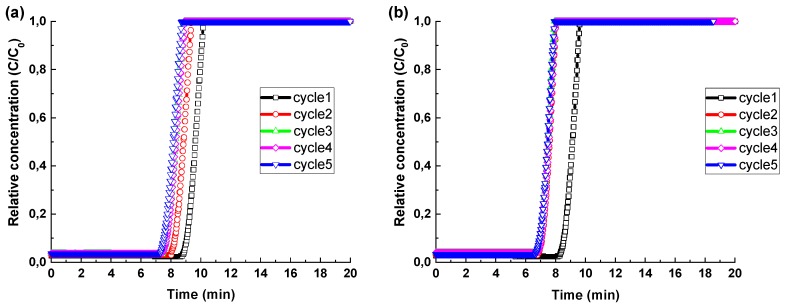
CO_2_ cyclic breakthrough measurements on (**a**) NaX granules; (**b**) CaA granules (□ black—cycle 1, ○ red—cycle 2, △ green—cycle 3, ◇ pink—cycle 4, ▽ blue—cycle 5).

Figure 5 shows the cyclic breakthrough experiments on NaX and CaA granules to determine the adsorption and mass transfer kinetic parameters. In a breakthrough curve, C/C_0_ represents the relative concentration of CO_2_, where C and C_0_ correspond to the concentration at the outlet and the inlet of the fixed bed, respectively. The relative concentration of zero in the breakthrough curve means that all the CO_2_ gas molecules are being adsorbed, whereas the relative concentration of one implies that the saturation of the bed has been achieved. The zone between zero to one is described as the mass transfer zone, where most of the mass transfer takes place [51]. Both curves offer the same breakthrough width, which is defined as the time difference to attain 5% and 95% of the final concentration of CO_2_, which implies that the gas velocity is uniform in both the columns containing NaX and CaA granules [52]. Stability was achieved after a few cycles of the breakthrough experiment as shown in Figure 5a,b. The CaA granules were able to attain stability faster than the NaX granules. The reduction in the breakthrough point was observed on both curves after cycle 1 due to the presence of unreleased chemisorbed CO_2_ even after performing regeneration by evacuation. The amount of the CO_2_ gas adsorbed by NaX and CaA was 5.7 mmol/g and 5.1 mmol/g, respectively, for cycle 1, and it reduced to 4.9 mmol/g and 4.2 mmol/g for the NaX and CaA granules, respectively, in cycle 5. This reduction in capacity with cycles can be attributed to the chemisorbed CO_2_ in the granules that were not able to evacuate completely or to an inefficient evacuation technique. The CO_2_ uptake rate for the stable cycle was 3.6 mg of CO_2_/g/s and 3.1 mg of CO_2_/g/s for NaX and CaA granules, respectively. The CO_2_ adsorption rate was higher than the one previously reported for NaX and CaA binderless beads [43]. Additionally, the granules were intact without any breakage after 5 adsorption-desorption cycles at 4 bar; this further enhanced the usability of freeze granules in the PSA for CO_2_ separation.

Breakthrough curves in Figure 5a,b have a sharp mass transfer zone that implies that these granules offer low mass transfer resistance with even gas flows within the column. The mass transfer coefficient was evaluated using the Klinkenberg Equation (2).

(2) $$C/C_{0}=1/2\times erfc\;\left(7\sqrt{\xi }/8-9\sqrt{\tau }/8\right)$$

where ξ and τ are defined as the dimensionless length and dimensionless time given by Equations (3) and (4), respectively. Parameter κ is the mass transfer coefficient, *K* is Henry’s law constant of the adsorbate, *z* is bed length, ϵ is total void fraction, *t* is time in minutes, and *υ* is interstitial velocity given by Equation (5), where *Q_V_* is the volumetric flow rate of the gas and *S* is the cross-sectional surface area of the bed.

(3) ξ=κKz((1−ϵ)/υϵ

(4) τ=κ(t−z/υ)

(5) $$\upsilon =Q_{V}\epsilon /S$$

Equation (2) was fitted to the experimental breakthrough data to simulate values of mass transfer coefficients. The mass transfer coefficient (κ) is 1.3 and 1.6 m/s for the NaX and CaA granules, respectively, higher than the one previously reported for a freeze-cast zeolite A monolith [53]. The mass transfer coefficient of freeze-granulated NaX and CaA is 29% and 166% higher than of the commercial binderless zeolite NaX and CaA granules (see supplementary information), respectively, as reported in our previous work. The higher mass transfer coefficient for CaA granules is due to the presence of large macropores created by ice-templating as confirmed from SEM and mercury intrusion porosimetry. However, these large networks of voids in the CaA granules may limit the available surface area and pore volume for the fluid to reach sufficient adsorption capacities.

## 3. Materials and Methods

### 3.1. Chemicals and Procedures

Zeolites NaX and CaA were purchased as powders with a particle size of 2–4 µm from Luoyang Jianlong Chemical Industry Co., Ltd (Yanshi, Henan, China). Bentonite clay, polyethylene glycol (PEG, MW 300), and polyacrylic acid (PAA, MW 50) were purchased from Sigma-Aldrich Chemie GmbH (Buchs, Germany), Merck Schuchardt OHG (Hohenbrunn, Germany), and Polysciences, Inc. (Warrington, PA, USA), respectively.

**Figure 6:**
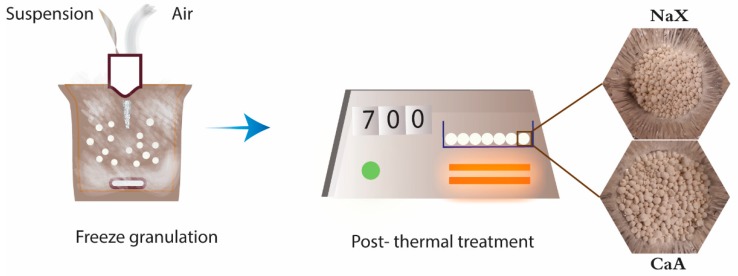
Schematic representation of the freeze granulation process with the resulting structured freeze granules.

The suspensions of zeolite NaX and CaA were prepared by dispersing 20 wt% of zeolite powder in 71 mL distilled water followed by magnetic stirring for 15 min. Bentonite clay (3 wt%) as an inorganic binder, water-soluble PEG (4 wt%), and PAA (2 wt%) as a sacrificial organic binder were added to the suspensions. The zeolite suspensions were placed on a magnetic stirrer overnight at room temperature for attaining homogeneous deagglomerated slurries. A PowderPro AB laboratory-scale granulator LS-6 (Sweden) equipped with a Watson–Marlow Bredel pump (England) was used for granulation. Figure 6 shows the freeze granulation process, including the resulting granules.

In the freeze granulation process, the homogeneous suspension was drawn by a suction tube using a pump at 30 rpm into a spray nozzle 0.7 mm in diameter. To avoid clogging of the spray nozzle by undissolved additives or agglomerates, the suspensions were subjected to continuous stirring at a rate of 500 rpm by a magnetic stirrer. The zeolite NaX and CaA suspensions were sprayed at an airflow of 0.19 bar and 0.17 bar, respectively, into the liquid nitrogen vessel to freeze the droplets instantaneously. The frozen granules were recovered in an aluminum tray and dried in a freeze dryer at −110 °C for 72 h under vacuum. After the freeze-drying process, the granules were subjected to the thermal treatment at a heating rate of 1°C/min up to 700 °C. The thermal treatment was carried out in a N11/HR/P300 (Nabertherm GmbH, Germany) annealing and hardening furnace with a temperature control accuracy of ±1 °C and temperature uniformity of ±5 °C. This thermal treatment was performed to remove temporary additives, such as PEG and PAA, and to strengthen the granules with the inorganic bentonite binder.

### 3.2. Characterization

The pH of the suspensions was measured using an HI 1110 Hanna benchtop pH meter (Woonsocket, RI, USA) prior to the freeze granulation process.

The viscosity measurements for aqueous NaX and CaA suspensions (prepared as described above) were performed at 293 K using a Discovery Hybrid Rheometer DHR-2 (TA Instruments, New Castle, DE, USA) using standard rotational mapping geometry with a 20 mm parallel Peltier plate. Prior to the measurement, the suspensions were stirred overnight for attaining homogenous mixtures and to avoid sedimentation. The shear rate varied from 20 up to 1000 s^−1^ for NaX and CaA rheology measurements.

Powder X-ray diffraction patterns were recorded for as received NaX and CaA zeolite powders and structured granules over the 2θ range between 5° to 70° with a step size of 0.02° using a PANalytical Empyrean instrument (Malvern, UK) equipped with a PIXcel 3D detector using Cu Kα radiation (wavelength 0.154 nm) at 40 kV and 45 mA. The investigated materials were grounded in a mortar and mounted on a stainless steel sample holder.

Scanning electron microscope micrographs were obtained using a JEOL JSM-IT300 microscope (Tokyo, Japan) operating at the acceleration voltage of 15 kV. The granules were crushed into powders and spread over a carbon tape. For obtaining micrographs of whole granules, a silver paste was used to hold granules on the sample holder. All the samples were subjected to gold sputtering before mounting on the sample holder to avoid any radiation damage and charging effect.

The surface area of the granules was determined using a micrometric Gemini VII 2390 Surface Area Analyzer (Micromeritics, Norcross, GA, USA) in the relative pressure (p/p_0_) range of 0.05–0.35 using the Brunauer–Emmett–Teller adsorption theory. Prior to the experiments, the samples were degassed under high vacuum (1 × 10^−4^ Pa) at 573 K overnight. The textural properties of the structured zeolite granules were evaluated using N_2_ adsorption experiments. The N_2_ adsorption experiments were carried out at 77 K using liquid nitrogen. Apart from the surface area determination, CO_2_ and CH_4_ adsorption isotherms were collected at 293 K and 273 K up to 100 kPa pressure for all the samples. The temperature was maintained in an isothermal Dewar flask using an external thermometer.

Mercury intrusion porosimetry was carried out using a Micromeritics AutoPore III-9400 instrument (Norcross, GA, USA) across a wide pressure range (0.03–420 MPa) to access the mesopores and macropores. The pore size distribution was estimated using the Washburn Equation (6), which describes the relationship between the pressures needed to intrude mercury into the capillary pores of radius *r*, where γ is the surface tension of 0.485 N/m and *θ* is the contact angle of mercury of 130°.

(6) p=−2γcosθ/r

Breakthrough experiments were performed on the freeze-granulated zeolites using a Pressure Swing Adsorption PSA-300LC instrument (L&C Science and Technology, Hialeah, FL, USA). The CO_2_ separation performance was investigated at 293 K and 4 bar for up to 5 cycles. The flow rate was set to 75 mL/min and 60 mL/min for CH_4_ and CO_2_ gases, respectively, and the binary mixture was passed through a 100 mm long and 15 mm wide fixed bed. Prior to the breakthrough experiments, the samples were regenerated at 473 K under the inert helium flow of 20 mL/min for 12 h. To ensure significant drying of the granules, the dew point of −18 °C was used as the standard. The amounts of the NaX and CaA freeze granules loaded in the bed were 4.4 g and 4.6 g, respectively. The breakthrough curves were determined by setting up relative concentration (C/C_0_) as the vertical axis and time as the horizontal axis, where C stands for the outlet concentration, and C_0_ is the initial concentration of the gaseous mixture.

## 4. Conclusions

NaX and CaA zeolite powders were structured into the hierarchically porous granules by optimizing the freeze granulation process parameters and suspension rheology to display efficient CO_2_ separation performance. Stable colloidal suspensions of zeolites were prepared with the optimum amount of bentonite featuring weak shear thinning rheological behavior to obtain the deagglomerated and feasible flow required for the freeze granulation procedure. The freeze granulation process yielded homogenous granules of about 2–3 mm in diameter without affecting zeolite crystallinity and morphology. Furthermore, pressureless thermal treatment was carried out to remove temporary additives and improved the mechanical stability of the granules to withstand rapid pressure swings. The NaX granules show the CO_2_ uptake of 5.8 mmol/g at 273 K, 45% higher than the CaA granules. The sharp front in the adsorption-desorption cyclic breakthrough experiment implies that the granules offer low mass transfer resistance. The NaX granules showed high CO_2_-over-CH_4_ selectivity of 214 as compared to the CaA granules as calculated using the Henry’s law. The mass transfer kinetic study was carried out by fitting the experimental breakthrough data in the Klinkenberg equation. The mass transfer coefficient of NaX granules was 1.3 m/s and is higher than of the commercially available binderless X zeolite granules due to the creation of extra macroporosity by ice-templating in the freeze granulation process, making it an efficient structured material for a PSA unit in the biogas upgrading process with a fast uptake rate of 3.6 mg of CO_2_/g/s.

## Figures and Tables

**Table 1:** A summary of textural properties of freeze-granulated zeolites and powders.

Sample Name	BET Surface Area (m^2^/g)	Micropore Area (m^2^/g)	External Surface Area (m^2^/g)	Micropore Volume (cm^3^/g)
CaA powder	528	441	86	0.21
CaA granules	411	351	60	0.18
NaX powder	685	644	41	0.35
NaX granules	557	513	43	0.25

## Supplementary Materials

The following are available online, Figure S1: CO_2_ breakthrough measurements on the commercial granules.
